# The yield of screening symptomatic contacts of multidrug-resistant tuberculosis cases at a tertiary hospital in Addis Ababa, Ethiopia

**DOI:** 10.1186/s13104-015-1442-z

**Published:** 2015-09-29

**Authors:** Addisalem Titiyos, Degu Jerene, Fikre Enquselasie

**Affiliations:** School of Medicine, Addis Ababa University, P.O. Box 54 code 1251, Addis Ababa, Ethiopia; Heal TB, Management Science for Health, Addis Ababa, Ethiopia; School of Public Health, Addis Ababa University, Addis Ababa, Ethiopia

**Keywords:** Contact tracing, MDR-TB, Ethiopia

## Abstract

**Background:**

Early detection and treatment of multidrug-resistant tuberculosis (MDR-TB, resistant to isoniazid and rifampicin) is an urgent global priority. Identifying and tracing close contacts of patients with MDR-TB could be a feasible strategy to achieve this goal. However, there is limited experience with contact tracing among patients with drug-resistant tuberculosis both globally and in Ethiopia. Here we present our findings on the extent of screening symptomatic contacts and its yield in a tertiary hospital in a major urban setting in Ethiopia.

**Results:**

Symptomatic household contacts were identified in 29 (5.7 %) of 508 index cases treated at the hospital. There were a total of 155 family members in the households traced of whom 16 (10 %) had confirmed MDR-TB. At least one confirmed MDR-TB cases was identified in 15 (51.7 %) of the 29 traced households.

**Conclusions:**

Tracing symptomatic contacts of MDR-TB cases could be a high yield strategy for early detection and treatment of MDR-TB cases in the community. The approach should be promoted for wider adoption and dissemination. Larger scale studies should be done to determine its effectiveness and sustainability in similar settings.

## Background

Multidrug-resistant tuberculosis (MDR-TB, resistant to isoniazid and refampicin) like drug-sensitive tuberculosis (TB) is transmitted through air droplets from infected person and they have a high potential to spread within people who have close contact with infected persons. Close contacts of MDR-TB patients are defined as people living in the same household, or spending long hours a day together with the patient in the same indoor living space. According to the World Health Organization (WHO), the International Union Against Tuberculosis and Lung Disease (IUATLD) and the International Standards of TB Care (ISTC), contacts of patients with multi or extensively drug-resistant TB (MDR/XDR-TB, XDR-TB is MDR-TB and have additional resistance to one of fluoroquinolones and any of the second-line injectables which include kanamycin, amikacin and capreomycin) should be closely followed up to prevent further spread of the bacteria [1, 2]. Close contacts of tuberculosis cases, such as household members, are the most likely to become infected due to intense and/or prolonged exposure to index cases in the weeks to months before diagnosis and treatment initiation [3].

In the absence of molecular epidemiologic data, secondary cases of MDR-TB within a household in an area with increasing incidence of MDR-TB are generally assumed to be the result of within-household transmission [4]. The spread of tuberculosis occurs mainly in settings where prolonged contact between people promotes the transmission from an infectious ‘index case’ with TB disease to one or several ‘contacts’. Contact tracing in general is believed to serve two functions: (1) identifies contacts with TB disease so that treatment can be initiated early when disease is more limited—this also serves to reduce transmission and (2) identifies high risk infected contacts who might benefit from either preventive therapy or close observation [5].

As is in many high MDR-TB burden countries, there is little experience with contact tracing of MDR-TB patients in Ethiopia. The main objective of this study was to assess the extent of screening symptomatic contacts in specialized tuberculosis treatment center in Addis Ababa. In this report, we present data on screening symptomatic contacts and its yield at a tertiary TB care center in Addis Ababa. The findings from this study are believed to inform the national MDR-TB treatment implementation plan as well as other similar countries in their effort to roll out MDR-TB treatment services.

## Methods

### Setting and study design

We conducted this study at St. Peter Tuberculosis Specialized Hospital (SPTSH) from February 2013 to April 2013 in Addis Ababa, Ethiopia. SPTSH was the first hospital to start MDR-TB treatment in 2009. The program was initiated as part of a pilot program with the Green Light Committee (GLC) approval to treat 45 patients. As of November 2012 there were over 508 patients enrolled in the MDR-TB care unit of the hospital [6]. Considering the total treatment period and to see the treatment out-come, we carried out retrospective chart and register review of patients enrolled and treated at SPTSH during the period February 2009–December 2012 to determine the yield and extent of household contact investigation.

### Screening contact practice at the hospital

In the hospital, it was a routine work practice to ask all index cases if any household member had respiratory symptoms suggestive of TB. If any symptomatic household members were identified, the index case was encouraged to bring the household member for further evaluation at the hospital. The clinic staff did thorough clinical evaluation of the symptomatic household member including detailed history, physical examination, and laboratory work up as per the national algorithm. Close contacts with no active TB disease were monitored carefully for at least 2 years. In particular, careful and close follow-up was encouraged for infants and children under 5 years of age. Those contacts with no signs and symptoms suggestive of active TB were educated about the signs and symptoms of TB, about their contact with an MDR-TB index case and about the importance of seeking treatment urgently if they develop signs and symptoms of TB disease. Follow up monitoring was done every 1–2 months. For contacts from Addis Ababa and nearby towns, community team members composed of a health officer and a nurse did 1–2 monthly home visits. Those contacts who came from outside Addis Ababa were encouraged to visit the clinic every 1–2 months.

### Data collection

We used secondary data abstraction form for data collection. We did data collection at two stages—first for the index cases and then for the contacts. For each index case we used the MDR-TB register as data source. The register contained the following variables: age, gender, marital status, employment status, whether the patient had MDR or XDR-TB, vital status within the last 24 months (as alive or dead), HIV status, and whether contacts were traced/screened. For contacts who were screened, detailed information was recorded in a separate Contact Tracing Form. Data recorded in the Contact Tracing Form included a list of all household members and their age, sex, symptoms, physical findings, HIV status, sputum microscopy, chest X-ray, and actions taken. Two health officers and four nurses who were working at the MDR-TB care unit collected the data by reviewing each patient’s chart and register of patient files at the MDR-TB centers. A two-day training was given for all data collectors. Data quality was controlled through continuous supervision by one of the authors (AT) during data collection. All completed data collection forms were examined for completeness and consistency during data management, storage and analysis.

### Data entry and analysis

We used EPI-INFO version 3.3.1 and SPSS version 16.0 for data entry and analysis respectively. A descriptive analysis was performed by calculating proportions. The median and inter-quartile- range were calculated to measure variability of quantitative variables. Results were analyzed with the outcome being whether contact tracing was performed. Categorical variables were compared using the χ^2^ test. *Odds ratios* (OR) and confidence intervals to 95 % (CI) were calculated as a measure of association. The variables found to have a *p* value ≤0.2 on a bivariate analyses were further analyzed using the logistic regression, step wise technique. A *p*-value of <0.05 was considered statistically significant.

### Ethical considerations

The study was approved by the Institutional Review Board (IRB) of the College of Health Sciences, Addis Ababa University. Following the approval, official letter of co-operation was written to St. Peter TB specialized hospital by the School of Public Health. The ethical committee of St. Peter TB specialized Hospital reviewed the protocol and agreed on the study. Since the study was conducted through review of medical records, no invasive procedures were involved. To preserve the confidentiality, nurses and health officers working in MDR-TB clinic of the hospital extracted the data from the medical records. Moreover, no personal identifiers were used on data collection form. The recorded data was not accessed by a third person.

### Operational definitions

Index case—the initially identified case of MDR-TB.

Contact case—a person who shared the same enclosed living space.

## Results

### Baseline information

We reviewed the records of 508 index cases. Their median age (interquartile range, IQR) was 27 years (23–35). Over a half (52 %) were men, 54 % were married and 41 % had secondary level of education. Only four (0.8 %) out of 508 index cases had history of previous exposure to confirmed MDR-TB or TB patient and majority of index cases were retreatment patients that received treatment either of first line anti TB drugs of WHO treatment category regimen previously. In the study population, 410 (80.7 %) were HIV negative, ninety eight (19.3 %) of confirmed MDR-TB index cases were also HIV positive, 87 (88.8 %) of whom started ART including four on second line ART regimens (Table 1).

**Table 1:** Baseline and socio demographic characteristics of index cases, St. Peter TB Specialized Hospital, 2013

Characteristics	Number (n = 508)	%
Age group		
<15	10	2
15–24	162	31.9
25–34	198	39
35–44	70	15.6
45+	59	11.7
Sex		
Female	244	48
Male	264	52
Marital status		
Single	287	56.5
Married	200	39.4
Undocumented	21	4.1
Educational level		
No formal education	44	8.7
Formal education	380	84.7
Undocumented	34	6.7
Exposure to MDR-TB patient		
Yes	4	0.8
No	504	99.2
Events		
Cured	93	18.3
On follow up	321	63.2
Drop	20	3.9
Died	53	10.4
Undocumented	21	4.1
HIV status		
Positive	98	19.3
Negative	410	80.7
Index cases traced		
Yes	29	5.7
No	479	94.3

### Characteristics and yield of contacts screened in the household

A symptomatic household contact was identified in 29 of 508 (5.7 %) index cases. Household screening and follow up was undertaken in these 29 symptomatic contacts. At least one confirmed MDR-TB case was identified in 15 of the 29 symptomatic contact traced households. The household contacts of the index cases were identified via the medical records of the index cases and through interviews; symptomatic contacts or family members identified on the screening form and attached with the respective index case file. Of 155 household contacts screened, 16 (10.3 %) were found to have MDR-TB. Of the 16 confirmed cases, 15 had already been started on treatment at the time of chart review; eight have shown improvement, three died, two of them were HIV positive and the outcome of five patients were not documented. The family size in the traced households ranged from 2 to 14. Nine (6 %) of the screened household contacts had previous history of TB treatment and four of the sixteen confirmed MDR-TB contact cases had previous history of TB treatment. From the sixteen confirmed MDR-TB contact cases, 13 (81.25 %) were also diagnosed for pulmonary TB (Table 2).

**Table 2:** Characteristics of index cases for whom contacts were identified, St. Peter TB Specialized Hospital, 2013

Characteristic	Number (n = 29)	%
Age group		
14–24	16	55.2
25–34	9	31
35–44	3	10.3
45+	1	3.4
Sex		
Female	20	69
Male	9	31
Familial position of the index case		
Mother/father	2	6.9
Sister/brother	12	41.4
Wife/husband	7	24.1
Child	4	13.8
Cousin	4	13.8
Place of living		
Addis Ababa	14	48.3
Out of Addis Ababa	15	51.7
Number of MDR-TB cases per household		
One	14	48.3
Two	12	41.4
Three	3	10.3
Number of symptomatic contacts who developed MDR-TB per household		
One	14	44.8
Two	1	3.4
None	14	44.8

### Factors associated with developing MDR-TB

The number of contacts traced for MDR-TB was too small to identify associated factors. However, we identified some degree of associations on univariate analyses. The odds of developing MDR-TB was five times [OR: 5, 95 % CI: 1.03, 24.279], higher among contacts from Addis Ababa as compared to the odds of contacts from other regional towns. Similarly the odds of developing MDR-TB was five times higher among contacts that received previous TB treatment [OR: 5.3, 95 % CI: 0.86, 32.02] as compared to those who didn’t receive previous TB treatment. From the confirmed contacts of MDR-TB, the odds of developing MDR-TB was 0.33 less likely among HIV positive contacts as compared to HIV negative [OR: 0.33, 95 % CI: 0.06, 1.74]. Sex of contacts compared on bi varate among contacts confirmed MDR-TB and the odds of female was three times higher compared to the odds of male [OR:3, 95 % CI: 0.58, 15.61].

On multivariate analyses none of the variables found to be statistically significant.

## Discussion

In this study, we found a high rate of confirmed MDR-TB cases among symptomatic household contacts of MDR-TB index cases. ONE IN TEN of the family members in the traced households had MDR-TB. The overall rate of contact tracing, however, was low and it focused on the symptomatic ones only. The study suggests that active tracing of symptomatic contacts of index MDR-TB cases could contribute to prompt identification and treatment of MDR-TB cases. This could be a highly effective strategy in saving more lives as well as in cutting the chain of the transmission in the community. Many risk factors for the development of MDR-TB have been reported among contacts. In our study, we considered variables such as place of living, previous history of TB treatment, HIV status, sex, age, number of confirmed MDR-TB in the house, and number of family traced.

As genetic studies were not performed, this study could not ascertain whether or not the source of infection was the index case. However, there is considerable evidence to support human-to-human MDR-TB strain transmission. Indeed over half of global MDR-TB cases are thought to result from primary transmission [7]. Moreover, our finding is similar to findings from a cross sectional study conducted in India among contacts of MDR-TB patients which showed high proportion of MDR-TB cases among contacts of MDR-TB index cases [8].

Although studies have shown that household contacts with TB are likely to have acquired infection independently in high-incidence settings, there are no published estimates of the probability that two household members with multidrug-resistant TB share a similar genotype and are members of the same transmission chain. Molecular epidemiologic data from households with more than one MDR-TB case can help shed light on the transmissibility of highly drug-resistant disease and also help guide public health policy. For example, international guidelines for the management of known contacts of MDR-TB patients recommend an empirical drug regimen based either on the drug-resistance profile of an isolate from the suspected index MDR-TB case-patient or on the most common drug-resistance pattern in the community while drug sensitivity tests are pending [9, 10]. Since this is the first report of the yield of MDR-TB contact investigation from Ethiopia and among few from the developing world, it provides useful information that can serve us input for planning contact investigation at larger scale.

The high rate of MDR-TB cases among traced household contacts suggests the need for improved TB control measures. The data calls for improved infection control measures, implementation of rapid diagnostics, and enhanced active screening strategies. This was suggested by others as well. A cross sectional study conducted in India among contacts of MDR-TB patients, for examples, showed from the total 302 contacts of 58 index MDR-TB patients traced 16 (5.29 %) developed TB and two (0.66 %) had MDR-TB. The study concluded that evaluation of contacts of MDR-TB case may lead to early diagnosis and prevention of tuberculosis [11].

Few studies have examined the burden of active disease in close contacts of MDR-TB patients [12–14]. A Brazilian study reported that the prevalence of TB infection and progression to active TB was comparable in close contacts of MDR-TB and drug-susceptible TB patients, despite the longer duration of exposure of contacts in patients with MDR-TB. Another study by Ottmani S et al. showed high proportion of index case contacts developed tuberculosis and the authors concluded that performing contact investigation as a routine activity of the national TB programme was feasible and useful in low–middle-income countries [15].

Whether only symptomatic contacts could be screened as a first stage in scaling up contact tracing in low and middle income countries is an possible consideration arising from our study. This would make contact tracing more feasible in resource limited settings given the burden of disease. In the systematic review by Shah et al. 8 % of household MDR-TB contacts were found to have MDR-TB. In our study 10 % of symptomatic contacts had MDR-TB. These figures are comparable but have different entry points as most of the studies included in the systematic review/meta analysis included screening of all MDR-TB contacts, not just those with symptoms. It may be that it is only necessary to screen symptomatic contacts [16].

Earlier diagnosis of MDR-TB remains a significant programmatic objective because of in this setting where close contacts of MDR-TB cases, such as household members, are the most likely to become infected, due to intense and/or prolonged exposure to index cases in the weeks to months before diagnosis and treatment initiation. Our study highlights the high proportion of MDR-TB in household contacts of MDR-TB cases. Dhingra et al. reported a 53.5 % prevalence of TB infection of disease in household contacts in their study group compared to 44 % in the general population [17]. A better understanding of the relative importance of intra household or community transmission may help to inform the choice of empirical regimens [18].

There are several limitations in the study. First, the small sample size of drug-resistant contact cases available for analysis of associated factors and contacts with active TB (only with cough symptom) did not allow for making valid conclusions as to factors associated with MDR-TB among household contacts. Second, data on several determinants for MDR-TB disease were absent from analysis because they were not in the routine registers and charts of the patients. Third, we considered only household contacts and not other casual or close contacts. Fourth, the investigation considered only recorded household contacts, were not able to find each household contact. Finally, the lack of molecular typing data which could help determine whether the drug susceptibility profiles between index and contact cases were from strains with the same genotype or not.

## Conclusions

Tracing symptomatic close contacts of MDR-TB cases could be a high yield strategy for early detection and treatment of MDR-TB cases in the community. The approach should be promoted for wider adoption and dissemination. Larger scale studies should be done to determine its effectiveness and sustainability in similar settings.
